# The Gelatin Treatment of Hæmoptysis

**Published:** 1903-03-07

**Authors:** 


### THE GELATIN TREATMENT OF HÆMOPTYSIS.

THE value of gelatin as a hæmostatic is scarcely open to question, but its general adoption for this purpose has not yet come about on account of the unpleasant effects and dangers which have followed its administration by subcutaneous injection. Dr. Tickler[1] is of opinion that by means of rectal injection these drawbacks can be prevented, and that when the drug is applied in this manner its efficacy in stopping hæmorrhage is not impaired. Although morphia is useful and effectual in some cases of hæmoptysis, there are others in which its use is fraught with danger, owing to the fact that it increases the tendency to suffocation by retarding the expulsion of blood from the bronchi. The treatment by morphia, moreover, gives no security for the final arrest of the hæmorrhage, and in the severer degrees of hæmoptysis it fails entirely. The drawbacks to the subcutaneous injection of solutions of gelatin are considerable. In the first place the procedure is accompanied with severe pain. Even if a local anæsthetic be employed the pain usually supervenes shortly after the operation, and may persist for two or three hours, and when the administration has to be repeated several times on successive days the painfulness of the procedure becomes a matter of serious consideration. Again, necrosis of the skin at the place of injection not infrequently occurs. In some cases tetanus has ensued, and samples of gelatin have been found to contain tetanus spores. The subcutaneous injection also causes fever. The introduction of gelatin per rectum is painless and free from danger, while it has the same effect as when given subcutaneously. It does not cause any action of the bowels. The solution is prepared by dissolving 50 grammes of gelatin in 1¼ litres of hot water and boiling gently for one hour, when the volume is reduced by evaporation to one litre. A 5 per cent. solution is thus obtained. It is then cooled down to the body temperature and a quarter of a litre (about 9 ounces) is slowly passed into the rectum by means of an indiarubber tube with a nozzle. The injection is given three times a day until the sputum ceases to show any trace of blood. The question as to whether prolonged boiling destroys the value of gelatin as a hæmostatic may be answered in the negative.

[1] Lancet, Feb. 28.

